# Some Uses of Sepia

**Published:** 1885-07

**Authors:** W. E. Leonard


### SOME USES OF SEPIA.

W. E. LEONARD.

In a brief paper on "Sepia," in the transactions of the Hahnemannian Medical Association, of Iowa (1883 and 4), Professor Cowperthwaite declares it to be "one of the most valuable, and at the same time most neglected, remedies of our materia medica."

Admitting that it may oftentimes have been overlooked by me from lack of knowledge of its uses, I wish to recount the following instances where it has done excellent service:

I. Several times it has acted like magic in the last months of pregnancy, when the mothers were tortured by the violent motions of the fœtus and were nervous and sleepless; and in most instances the women have been those worn out by too-frequent child-bearing, and thin, sallow, and of the nervous type of temperament.

*Psorinum*, clinically, of course, is indicated for a like condition, "with tympanitic abdomen;" also *Opium*. (Jahr.)

II. October 31st, 1881.—Mrs. C., mother of three children. "Old dyspepsia," constant pain in stomach, worse immediately after eating; making the whole abdomen sore around the back; worse afternoons; milk disagrees.

She is a tall, strong woman, generally in the best of health.

The indications were meagre enough, nor could more definite ones be elicited.

Referring to the Repertory, the following helped to find the medicine for the case:

Aggravation after eating (chief remedies): *Bry., Calc., Caust., Con., Kali-carb., Nat. m., Nux, Phos., Sep., Sil., Zinc.* Aggravation from milk: *Arg., Arsen., Calc., Chin., Cic., Con., Nitr. ac., Nux, Sep., Sulph., Zinc.*

Sore pains in lower parts: *Canth., Igt., Nux, Sep., Zinc.*

Here were the three legs of the stool to stand upon, and the choice lay between *Nux*, *Sepia*, and *Zinc*.

I knew that this lady was excessibly irritable, "ugly," her husband said, during her pregnancies. This decided for *Sepia*. It was given, a few powders of the $5^{m}$ potency, to stop as soon as relief came.

Relief came promptly, and now, several years having passed, she has had no return of those symptons.

This was not an aggravated *Sepia* case, with some eructations, excessive flatulence, etc., but with enough of the condition manifested to lead to the right remedy.

III. December 21st, 1880.—Mrs. B.—Since birth of her last child, four years ago, after extra muscular exertion she is awakened from sleep "always after midnight" by numbness and severe pain in the right arm, "as if the flesh was being torn off from the finger-nails upward"—lasting ten minutes to half an hour; the paroxysm is preceded by a full, bursting feeling in arm and hand. She had used all sorts of nostrums for relief, but found none.

Occasionally, after being in the cold air or washing in warm water, she suffered with severe pains about the joints of the fingers.

Menses every three weeks and scanty, preceded the day before by backache, bearing down, and cutting in the uterine region.

The patient has a dark, yellow skin, and is thin and wiry: ℞ Sepia$^{6m}$, d., and Sac-lac.

She was to report in a week.

Not until July 12th, 1881, did she appear in the office again. She stated that my medicine had removed all the severe pain in the arm and joints, but she still had occasionally a numbness in the right arm, and sometimes in both hands.

The menses came as before with a leucorrhœa between the periods. She had been through much hard work, and was much debilitated. Two doses of *Sepia*$^{6m}$ were given, to be taken twelve hours apart, and a bottle of Sac-lac.

Although her family has been treated many times since, I was not consulted by her again until this month, nearly four years ago, when *Sepia*$^{30}$ was given, with benefit, for symptoms of an acute cold on the chest. Her other troubles had quite disappeared, and she has grown fleshy and strong.

In the first prescription her menstrual symptoms, and the parts chiefly affected by the pain (joints of fingers, etc.), together with the aggravation from washing in water, led to the remedy.

